# Ex vivo modelling of PD-1/PD-L1 immune checkpoint blockade under acute, chronic, and exhaustion-like conditions of T-cell stimulation

**DOI:** 10.1038/s41598-021-83612-3

**Published:** 2021-02-17

**Authors:** Alexander Roberts, Lindsay Bentley, Tina Tang, Fay Stewart, Chiara Pallini, Joel Juvvanapudi, Graham R. Wallace, Alison J. Cooper, Aaron Scott, David Thickett, Sebastian T. Lugg, Hollie Bancroft, Bridget Hemming, Charlotte Ferris, Gerald Langman, Andrew Robinson, Joanne Chapman, Babu Naidu, Thomas Pinkney, Graham S. Taylor, Kristian Brock, Zania Stamataki, Catherine A. Brady, S. John Curnow, John Gordon, Omar Qureshi, Nicholas M. Barnes

**Affiliations:** 1 Institute of Clinical Sciences, College of Medical and Dental Sciences, University of Birmingham, Vincent Drive, Edgbaston, Birmingham, B15 2TT UK; 2 Celentyx Ltd, Birmingham Research Park, Vincent Drive, Edgbaston, Birmingham, B15 2SQ UK; 3 Institute of Inflammation and Ageing, College of Medical and Dental Sciences, University of Birmingham, Vincent Drive, Birmingham, B15 2TT UK; 4 Academic Department of Surgery, University Hospitals Birmingham NHS Foundation Trust, Queen Elizabeth Hospital Birmingham, Mindelsohn Way, Edgbaston, Birmingham, B15 2GW UK; 5 Institute of Immunology and Immunotherapy, College of Medical and Dental Sciences, University of Birmingham, Vincent Drive, Edgbaston, Birmingham, B15 2TT UK; 6 Diagnostics, Drugs, Devices and Biomarkers, Cancer Research UK Clinical Trials Unit, University of Birmingham, Edgbaston, Birmingham, B15 2TT UK; 7 MIDRU, Birmingham Heartlands Hospital, Bordesley Green East, Birmingham, B9 5SS UK

**Keywords:** Cancer, Immunology, Oncology

## Abstract

Blockade of PD-1/PD-L1 interactions is proving an exciting, durable therapeutic modality in a range of cancers whereby T cells are released from checkpoint inhibition to revive their inherent anti-tumour activity. Here we have studied various ways to model ex vivo T cell function in order to compare the impact of the clinically utilised anti-PD-1 antibody, pembrolizumab (Keytruda) on the activation of human T cells: focussing on the release of pro-inflammatory IFNγ and anti-inflammatory IL-10 to assess functionality. Firstly, we investigated the actions of pembrolizumab in an acute model of T-cell activation with either immature or mature allogeneic dendritic cells (DCs); pembrolizumab enhanced IFNγ and IL-10 release from purified CD4+ T-cells in the majority of donors with a bias towards pro-inflammatory cytokine release. Next, we modelled the impact of pembrolizumab in settings of more chronic T-cell activation. In a 7-day antigen-specific response to EBV peptides, the presence of pembrolizumab resulted in a relatively modest increase in both IFNγ and IL-10 release. Where pembrolizumab was assessed against long-term stimulated CD4+ cells that had up-regulated the exhaustion markers TIM-3 and PD-1, there was a highly effective enhancement of the otherwise exhausted response to allogeneic DCs with respect to IFNγ production. By contrast, the restoration of IL-10 production was considerably more limited. Finally, to assess a direct clinical relevance we investigated the consequence of PD-1/PD-L1 blockade in the disease setting of dissociated cells from lung and colon carcinomas responding to allogeneic DCs: here, pembrolizumab once more enhanced IFNγ production from the majority of tumour preparations whereas, again, the increase in IL-10 release was modest at best. In conclusion, we have shown that the contribution of PD-1—revealed by using a canonical blocking antibody to interrupt its interaction with PD-L1—to the production of an exemplar pro- and anti-inflammatory cytokine, respectively, depends in magnitude and ratio on the particular stimulation setting and activation status of the target T cell. We have identified a number of in vitro assays with response profiles that mimic features of dissociated cell populations from primary tumours thereby indicating these represent disease-relevant functional assays for the screening of immune checkpoint inhibitors in current and future development. Such in vitro assays may also support patient stratification of those likely to respond to immuno-oncology therapies in the wider population.

## Introduction

The blockade of inhibitory immune checkpoints has emerged as a transformative strategy to achieve durable anti-tumour responses in patients. Immune checkpoints contribute to the control of immune responses to prevent excessive tissue damage or autoimmunity. In the tumour environment however, these checkpoints can be subverted to prevent the immune system generating an effective anti-tumour response^1,2^. The use of ipilimumab, an anti-CTLA-4 blocking antibody was approved by the FDA in 2011 for the treatment of metastatic melanoma. This has been followed by blockers of the inhibitory PD-1/PD-L1 axis including the approvals of pembrolizumab (Keytruda) and nivolumab (Opdivo) in 2014. Now, checkpoint inhibition has been approved in multiple cancer types and numerous new agents are currently undergoing clinical trials.

However, there is variability in patient responses to PD-1 treatment depending on the cancer treated, with patients suffering from gastric cancer, head and neck squamous cell carcinoma and small cell lung cancer having an overall response-rate of 12–16% compared to those with melanoma or non-small cell lung cancer having an overall response rate of 26–55%^3^. Non-responsive patients or indeed induction of severe side effects often arising from over activation of the immune system highlight the continuing need for further therapeutic strategies. Understanding the cellular and molecular basis as to how checkpoint blockers work should help us to discern which patients may respond to these agents, reveal which agents may act most effectively in combination, and develop new therapeutics utilising additional mechanistic approaches for patient benefit.

Co-engagement of the T-cell receptor alongside appropriate co-stimulatory signals triggers a signalling cascade culminating in key T cell effector mechanisms, including cytokine release and cytotoxic function^4^. The careful regulation of T cell activation limits collateral damage from immune system activation; however, in the context of tumour immunity, inhibitory ligands and factors can be upregulated by tumour cells or other cells within the tumour microenvironment. Such inhibitory ligands (and mechanisms), of which PD-L1 is a key example, can limit the activation of T cells in this environment by delivering inhibitory signals through the PD-1 receptor and hence suppress T cell activation^5^.

Cytokines such as IFNγ and IL-10 play critical roles as humoral messengers for pro-inflammatory and anti-inflammatory signalling. For example, IFNγ is secreted by activated T cells and NK cells to promote anti-tumour immunity through: enhancing antigen presentation and pro-inflammatory differentiation of macrophages; stabilising Th1 lineage commitment, and; restraining regulatory T cell induction^6^. Furthermore, IFNγ can exert anti-proliferative, anti-angiogenic and pro-apoptotic effects on tumour cells directly. The archetypal suppressive cytokine, IL-10, can be produced by multiple cell types including regulatory T cells, Tr1 cells and macrophages^7^. IL-10 can suppress T-cell proliferation, T cell cytokine production, dendritic cell maturation, IgE and IgG4 production, and reduce the release of pro-inflammatory mediators from mast cells, eosinophils and basophils^8^. In cancer, the role of IL-10 is complex having both pro- and anti-tumoural effects^9^.

We have studied the impact of checkpoint inhibition on IFNγ and IL-10 release using primary human cells in a range of in vitro systems to identify straightforward models that could underpin the discovery and development of immuno-oncology therapeutics while also acting as potential predictors of individual patient responses.

## Materials and methods

### Generation of DC

Peripheral blood mononuclear cells (PBMC) were isolated from leukocyte cones (NHS-BTS) using Ficoll-Paque PLUS isolation. Monocytes were further enriched from PBMC using EasySep Human Monocyte Isolation Kit (STEMCELL Technologies, 19359), and CD14 purity verified by flow cytometry. For differentiation into dendritic cells (DC), monocytes were cultured at 0.5 × 10^6^/ml in RPMI-1640 with 10% heat-inactivated human serum (HI-HS) and Penicillin/Streptomycin (PS) (penicillin:100 U, streptomycin: 0.1 mg/ml; all Sigma-Aldrich), plus human (h) IL-4 (500 U) and hGM-CSF (1000 U) (both Immunotools) for 6 days. At day 2 cells were re-fed with hIL-4 (500 U) and h-GM-CSF (1000 U).

For generation of mature DCs (mDCs), after 6 days differentiation cells were harvested, counted and returned to culture at 0.5 × 10^6^/ml in RPMI-1640 with 10% HI-HS and 1% PS plus hIL-1β (1.0 μg/ml), hIL-6 (200 ng/ml), hTNFα (10 ng/ml) (all Immunotools), and hPGE_2_ (1.0 μg/ml, Sigma-Aldrich) for a further 2 days. For immature DCs (iDCs) cells remained in culture for the additional 2 days without any cytokines added. After 8 days both mDCs and iDCs were harvested, centrifuged at 400×*g* for 5 min and resuspended in 10% DMSO/90% HI-HS. DC’s were then stored long-term in the vapour phase of liquid nitrogen.

### Co-culture of T cells with allogeneic DCs

PBMC were isolated from leukocyte cones (NHS-BTS) using Ficoll-Paque PLUS isolation. CD4^+^ and CD8^+^ T cells were enriched (separately) from PBMC using CD4^+^ T cell Enrichment Kit and CD8^+^ T cell Enrichment Kit (STEMCELL Technologies, 19052, 19053 respectively). T cells were then stained with proliferation dye (ThermoFisher, 65-0842-85), washed with complete media [RPMI-1640 with 10% heat-inactivated fetal calf serum (HI-FCS) and Penicillin/Streptomycin (PS) (penicillin:100 U, streptomycin: 0.1 mg/ml; all Sigma-Aldrich)], (400×*g*, 5 min) for 3 washes in total. T cells were cultured with either: iDCs or mDCs at a ratio of 100,000 T cells: 10,000 DCs in the presence of α-PD-1 (pembrolizumab [Keytruda], 1.0 μg/ml, UHB Pharmacy), IgG4 isotype control (1.0 μg/ml, Biolegend, 403702), α-CTLA-4 (ipilimumab, [Yervoy], 1.0 μg/ml, UHB Pharmacy) or IgG1 isotype control (1.0 μg/ml, Biolegend, 403502) for 4 days. After 4 days culture, cells were stained with viability dye APC/Cy7 (BioLegend, 423106) plus either anti-human CD4 Pe/Cy7 (BioLegend, 300512) or anti-human CD8 FITC (BioLegend, 301006). Cells were then analysed using a cyAn ADP flow cytometer (Beckman Coulter). In addition, supernatants were analysed for IFNγ and IL-10 levels by ELISA (R&D Systems, DY285B, DY217B respectively).

### Dendritic cell phenotyping

iDCs and mDCs harvested at day 8 were analysed for phenotypic markers by flow cytometry. Cells were fixed using Human FoxP3 Buffer Set (BD Biosciences, 560098), and stained with anti-human: CD80 APC, CD86 BV510, HLA-DR AlexaFluor488, PD-L1 PE, (all BioLegend, 305220, 305431, 307656, 329705, respectively). Cells were then analysed using a cyAn ADP flow cytometer (Beckman Coulter).

### Generation of exhausted CD4^+^ T cells

PBMC were isolated from leukocyte cones (NHS-BTS) using Ficoll-Paque PLUS isolation. CD4^+^ T cells were further enriched from PBMC using CD4^+^ T cell Enrichment Kit (STEMCELL Technologies, 19052), and CD4 purity verified by flow cytometry. Cells were then cultured in a 24 well plate at a density of 1 × 10^6^/ml, in RPMI-1640 with 10% heat-inactivated foetal calf serum (HI-FCS) and 1% PS (referred to as complete media throughout). Cells were stimulated with phytohaemagglutinin (PHA; 5.0 μg/ml; Sigma-Aldrich) and hIL-2 (1000 U; Immunotools) for 14 days. Cultures were re-fed with hIL-2 (1000 U) at days 3, 5, and 8. At day 0 (unstimulated cells), and after 14 days culture, cells were harvested, centrifuged at 400×*g* for 5 min and resuspended in 10% DMSO/90% HI-HS. Cells were then stored long-term in the vapour phase of liquid nitrogen.

### Co-culture and cytokine measurements

CD4^+^ T cells from day 0 (unstimulated) and day 14 (PHA stimulated) were labelled with e450 proliferation dye (10 µM; ThermoFisher) and cultured with either: iDCs or mDCs at a ratio of 100,000 CD4^+^ T cells: 10,000 DCs in the absence (complete media) or presence of α-PD-1 (pembrolizumab, 1.0 μg/ml, UHB Pharmacy), IgG4 isotype control (1.0 μg/ml, Biolegend, 403702), α-CTLA-4 (ipilimumab, 1.0 μg/ml, UHB Pharmacy), or IgG1 isotype control (1.0 μg/ml, Biolegend, 403502) for 6 days. After 6 days culture, supernatants were analysed for IFNγ and IL-10 levels by ELISA (R&D Systems, DY285B, DY217B respectively). Cells were stimulated for 4 h with either brefeldin alone (10 µg/ml; Sigma-Aldrich), brefeldin + phorbol 12-myristate 13-acetate (PMA; 50 ng/ml; Sigma-Aldrich) or brefeldin + PMA + ionomycin (750 ng/ml; Sigma-Aldrich). Cells were then washed, surface labelled with anti-human CD4 BV510 (Biolegend; 317444), fixed and permeabilised using transcription factor staining buffer set (ThermoFisher, 00-5523-00) and labelled with anti-human IFNγ PE and IL-10 APC (Biolegend; 506507; 506807 respectively). Cells were then analysed using a Cytek flow cytometer.

### T cell phenotyping

Phenotypic markers expressed by CD4^+^ T cells isolated at day 0 and from day 14 were analysed by flow cytometry. Cells were fixed using Human FoxP3 Buffer Set (BD Biosciences, 560098), and stained with anti-human: CD4 BV510, LAG-3 Pe/Cy7, TIM-3 PE, TIGIT PeDazzle594, CTLA-4 BV421 (all BioLegend, 317444, 369310, 345006, 372716, 369606), CD3 FITC, and PD-1 APC (both ThermoFisher, 11-00390-42, 17-2799-42 respectively). Cells were then analysed using a cyAn ADP flow cytometer (Beckman Coulter).

### EBV peptide stimulation of PBMC

PBMC were isolated from a leukocyte cone (NHS-BTS) using Ficoll-Paque PLUS isolation. PBMC were then resuspended at 2 × 10^6^/ml and stimulated with EBNA-1 PepTivator (100 ng/ml, Miltenyi Biotech, 130-093-613) in the presence of α-PD-1 (pembrolizumab, 1.0 μg/ml, UHB pharmacy) or IgG4 isotype control (1.0 μg/ml, Biolegend 403702) for 7 days. After 7 days culture, supernatants were analysed for IFNγ and IL-10 levels by ELISA (R&D Systems, DY285B, DY217B respectively).

### Dissociated tumour cell experiments

Lung or colon tissue (obtained with appropriate Research Ethics Committee approval from consented patients at Heartlands Hospital or Queen Elizabeth Hospital) was initially minced before being digested in an enzyme cocktail containing: hyaluronidase, collagenase and DNAse (7.5 mg/ml, 1.0 mg/ml, 40 μg/ml respectively; all Sigma-Aldrich) for 1 h 15 min at 37 °C. The cell suspension was then washed through a 100 μm cell strainer with complete media, and then washed twice (400×*g* for 5 min) in complete media. Dead cells were removed from the cell suspension using a dead cell removal kit (Miltenyi-Biotec, 130-090-101). The live cell fraction was washed (400×*g* for 5 min) in complete media and resuspended at 2 × 10^6^/ml. Frozen mDCs were rapidly thawed at 37 °C, centrifuged at 400×*g* for 5 min in complete media and resuspended at 4 × 10^5^/ml. mDCs were cultured alongside the isolated live cell fraction at a ratio of 200,000 live cells:20,000 mDCs in the presence of α-PD-1 (pembrolizumab, 1.0 μg/ml, UHB pharmacy) or IgG4 isotype control (1.0 μg/ml, Biolegend 403702) for 2 days. After 2 days culture supernatants were analysed for IFNγ and IL-10 levels by ELISA (R&D Systems, DY285B, DY217B, respectively).

### Statistical analysis

Data are presented as mean ± SEM. A Shapiro–Wilk test was used to test normality. 2-tailed T-test, one sample T-test, one sample Wilcoxon test and Mann–Whitney U test were used to test statistical significance. Statistical analysis was performed using GraphPad Prism (version 8), and *p* < 0.05 was deemed statistically significant.

### Ethical statement

All samples were obtained with informed consent and with approval from the appropriate Research Ethics Committee. All research was carried out in accordance with relevant guidelines and regulations. All methods were approved by the relevant institution. Human colon cancer tissue from the Queen Elizabeth Hospital was obtained from consented patients via the Human Biomaterials Resource Centre (HBRC) under ethical approval (NRES Committee North West—Haydock; Ref 15/NW/0079). Human lung carcinoma tissue from Heartlands Hospital under national research ethical approval (REC 17/WM/0272).

## Results

### Effects of pembrolizumab on IFNγ and IL-10 levels in co-cultures of CD4^+^ T cells and allogeneic dendritic cells

**Figure 1:**
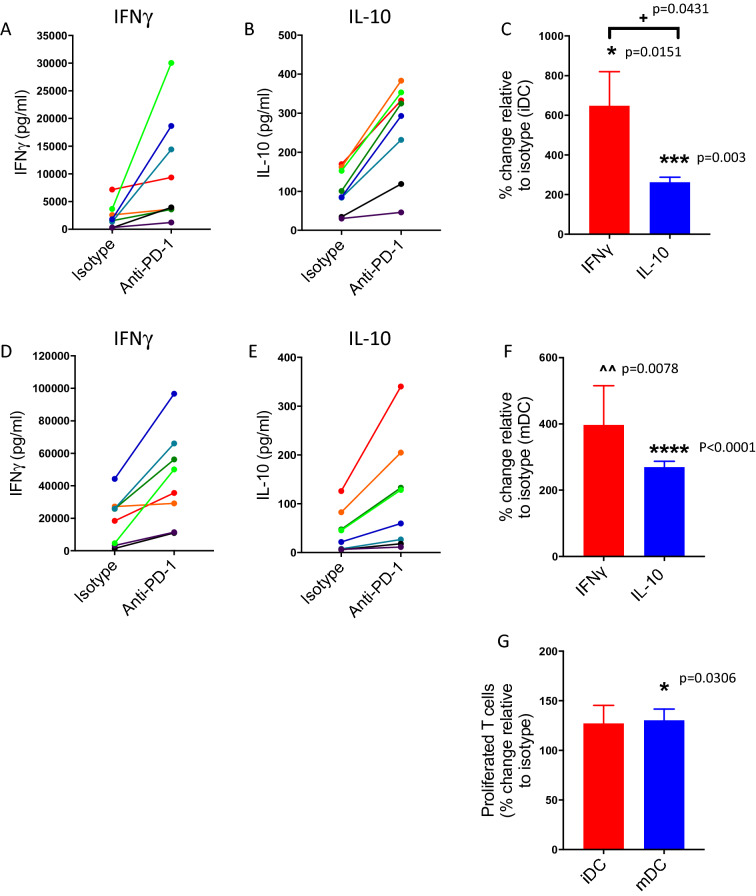
Pembrolizumab increases IFNγ and IL-10 levels in co-cultures of CD4^+^ T cells and allogeneic dendritic cells. Ability of pembrolizumab (anti-PD-1; 1.0 μg/ml) to modify IFNγ (**A**,**D**) or IL-10 (**B**,**E**) levels in co-cultures of CD4^+^ T cells and allogeneic immature (**A**–**C**) or mature (**D**–**F**) dendritic cells relative to isotype control. (**A**,**B**,**D**,**E**) indicate responses of individual donors (each colour represents an individual donor), (**C**,**F**) show percentage change in response in the presence of pembrolizumab relative to isotype control (mean + SEM, n = 8). (**G**) Shows impact of pembrolizumab upon CD4^+^ T cell proliferation in the presence of allogeneic immature or mature dendritic cells. Data presented as percentage change relative to isotype (mean + SEM, n = 8). The impact of pembrolizumab was statistically significant compared to isotype control (* one sample T-test or ^ one sample Wilcoxon test). There was a significant difference between IFNγ and IL-10 (+ 2-tailed T-test).

We initially tested the impact of pembrolizumab (Keytruda) on IFNγ and IL-10 production from purified CD4^+^ T cells cultured for four days with either immature or mature allogeneic dendritic cells (DCs). As anticipated, mature versus immature DCs expressed higher levels of HLA-DR, CD80, CD86 and PD-L1 on their cell surface (Supplementary Fig. S1 online) and evoked higher levels of IFNγ in the cell culture supernatant in comparison to immature dendritic cells (Fig. 1A,D). In the majority of donors, pembrolizumab increased the levels of IFNγ measured in the supernatant from CD4^+^ T cells cultured with either immature or mature DCs (Fig. 1A,D). Release of IFNγ by CD8^+^ T cells with DCs was also increased by the presence of pembrolizumab, although the magnitude of the change induced by immature DCs and pembrolizumab was smaller than that for CD4^+^ T cells (Supplementary Fig. S2 online). PD-1 blockade also enhanced IL-10 levels from cultures of CD4^+^ T cells and either immature or mature DCs although not to the same extent as the increase in IFNγ levels (Fig. 1B,E). There was a significant increase in cytokine production (both IFNγ and IL-10), relative to isotype control, for CD4^+^ T cells in the presence of mature or immature DCs with pembrolizumab (Fig. 1C,F). Under these stimulation conditions, changes in CD4^+^ T cell proliferation were not clearly evident in the presence of immature DCs. However, there was a significant increase (relative to isotype control) in CD4^+^ T cell proliferation in the presence of mature DCs and pembrolizumab (Fig. 1G). We also tested the impact of the CTLA-4-targetting antibody ipilimumab (Yervoy) on cytokine levels and proliferation and by contrast with pembrolizumab did not detect major changes under these assay conditions (Supplementary Fig. S3 online).

### Effects of pembrolizumab on IFNγ and IL-10 levels from PBMC cultures stimulated with EBV peptides

**Figure 2:**
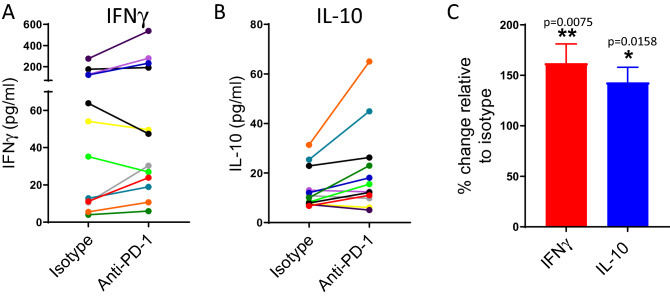
Pembrolizumab increases IFNγ and IL-10 levels in cultures of PBMCs stimulated with EBV peptides. Ability of pembrolizumab (anti-PD-1; 1.0 μg/ml) to modify IFNγ (**A**) or IL-10 (**B**) levels in cultures of PBMCs stimulated with EBV peptides. (**A**,**B**) indicate responses of individual donors (each colour represents an individual donor). (**C**) indicates percentage change in response in the presence of pembrolizumab relative to isotype control (mean + SEM, n = 12). The impact of pembrolizumab was statistically significant compared to isotype control (*one sample T-test).

We next wished to characterise the impact of PD-1 blockade in antigen-specific cells under more chronic conditions of stimulation. Here, PBMCs were stimulated with an EBV peptide pool for seven days and, again, IFNγ and IL-10 were measured in the cell culture supernatant. As expected, the levels of both IFNγ and IL-10 were much lower than when using allogeneic DCs as a stimulus (Fig. 2A–C). Using these stimulation conditions, pembrolizumab again increased levels of both IFNγ and IL-10 in the PBMC cultures, which was statistically significant when compared with isotype control (Fig. 2C). Overall there was a somewhat greater release of IFNγ when comparing individual donor responses. Thus, in this setting of ‘antigen- specific’ T cells, pembrolizumab again was capable of enhancing T cell responses.

### Impact of pembrolizumab on IFNγ and IL-10 levels in co-cultures of chronically stimulated T cells expressing exhaustion markers with allogeneic dendritic cells

**Figure 3:**
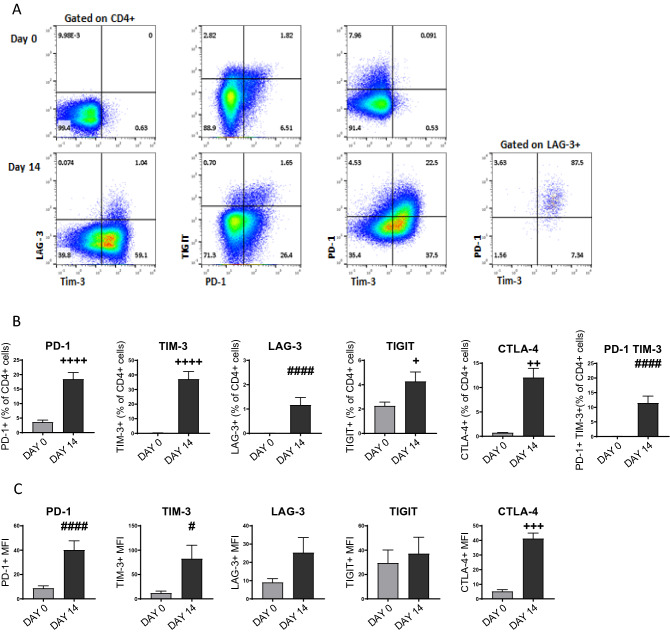
Exhausted CD4^+^ T cell phenotype. Representative (n = 6) flow cytometry plots (**A**) demonstrating expression of cell surface markers (LAG-3, PD-1, TIGIT, TIM-3) indicating an exhaustive cell phenotype on CD4^+^ T cells characterised at day 0 (unstimulated, upper panels) and after 14 days stimulated with PHA (5.0 μg/ml, lower panels). Impact of PHA (5.0 μg/ml)-induced exhaustion upon expression of cell surface markers (PD-1, TIM-3, LAG-3, TIGIT, CTLA-4) on CD4^+^ T cells. Data presented as percentage of expressing CD4^+^ T cells (**B**), and MFI of CD4^+^ T cells (**C**), (mean + SEM, n = 4–10). The impact of PHA treatment on surface marker expression was significant (+ 2-tailed T-test, # Mann–Whitney U Test).

In the tumour microenvironment, presumed as a result of chronic antigen exposure, T cells can show a dysfunctional or ‘exhausted’ phenotype. We therefore wished to assess the effect of pembrolizumab on cytokine production from cells that had previously been chronically activated. We first stimulated CD4^+^ T cells for 14 days with PHA, which increased the percentage (Fig. 3A,B) and MFI (Fig. 3C) of cells expressing PD-1, LAG-3, CTLA-4 and TIM-3; the number of TIGIT positive cells were not changed substantially in this model (Fig. 3A–C).

**Figure 4:**
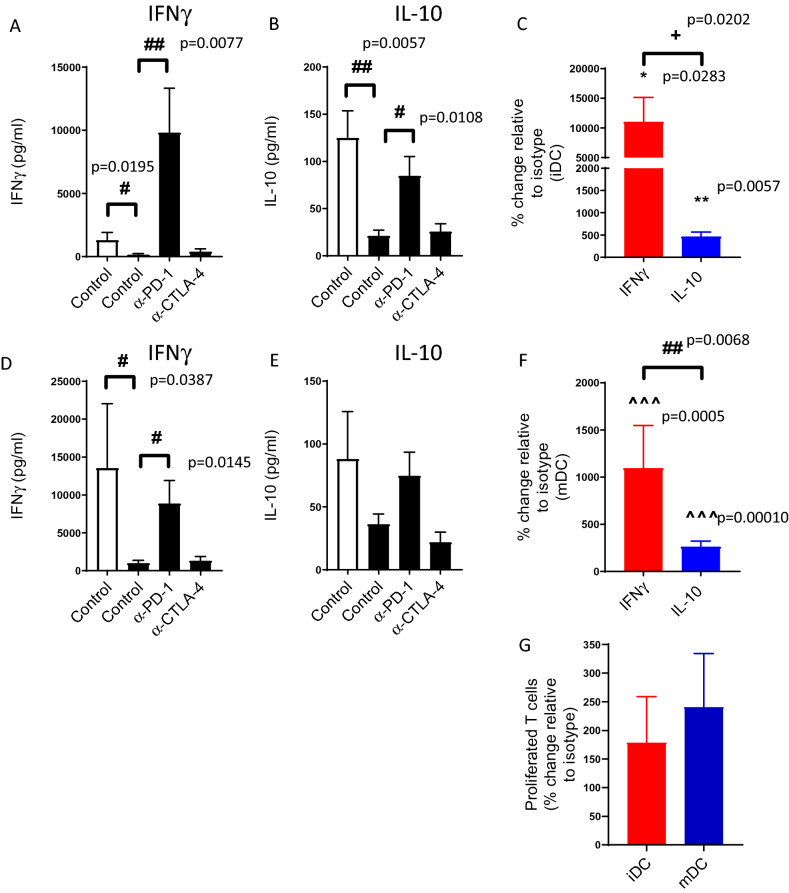
Exhausted CD4^+^ T cell function. Ability of pembrolizumab (α-PD-1; 1.0 μg/ml) and ipilimumab (α-CTLA-4; 1.0 μg/ml) to modify IFNγ (**A**,**D**) or IL-10 (**B**,**E**) levels in co-cultures of CD4^+^ T cells either unstimulated (clear bars) or pre-stimulated for 14 days with PHA (5.0 μg/ml; black bars) and allogeneic immature (**A**–**C**) or mature dendritic cells (**D**–**F**) relative to isotype control (mean + SEM, n = 9–13). (**C**,**F**) show percentage change in response in the presence of pembrolizumab relative to isotype control, (mean + SEM, n = 9–13). (**G**) Shows impact of pembrolizumab upon exhausted CD4^+^ T cell proliferation in the presence of allogeneic immature or mature dendritic cells. Data presented as percentage change relative to isotype (mean + SEM, n = 4). There was a significant difference between PHA-treated (exhausted) CD4^+^ T cells and unstimulated cells, and a significant difference between exhausted CD4^+^ T cells treated with isotype or treated with pembrolizumab (# Mann–Whitney U-Test). The impact of pembrolizumab was statistically significant compared to isotype control (*one sample T-test or **^**one sample Wilcoxon test). There was a significant difference between IFNγ and IL-10 (+ 2-tailed T-test, # Mann–Whitney U test).

On subsequent stimulation of these cells, with either immature or mature allogeneic DCs, the release of IFNγ and IL-10 was greatly diminished when compared with cells that had not undergone prior PHA stimulation (i.e. unstimulated, Fig. 4). Treatment of these chronically activated ‘exhausted’ CD4^+^ T cells with pembrolizumab enhanced substantively the IFNγ response of these cells when stimulated with allogeneic DCs (Fig. 4A,D). In the case of immature DCs, this enhancement of IFNγ levels was greater than was evident with the unstimulated CD4^+^ T cells. Whilst pembrolizumab also enhanced IL-10 levels in response to either immature or mature allogeneic DCs (Fig. 4B,E), the magnitude of this increase was considerably less than that seen for IFNγ release (Fig. 4C,F). For both immature and mature DCs, pembrolizumab induced a statistically significant increase in cytokine production (both IFNγ and IL-10) relative to isotype control (Fig. 4C,F). Therefore in this context, pembrolizumab skewed the cytokine response towards pro-inflammatory IFNγ over anti-inflammatory IL-10. Although the fold-increase was reduced compared to IFNγ, there was a trend for pembrolizumab to also enhance proliferation of these cells (Fig. 4G). In addition, to directly investigate the impact of pembrolizumab on IFNg and IL-10 production, we performed intracellular cytokine staining after re-stimulation with DCs. This revealed a trend towards pembrolizumab-enhancement of IFNg and IL-10 production (Supplementary Fig. S5 online).

### Effects of pembrolizumab on IFNγ and IL-10 levels in cultures of dissociated cells from solid tumours

**Figure 5:**
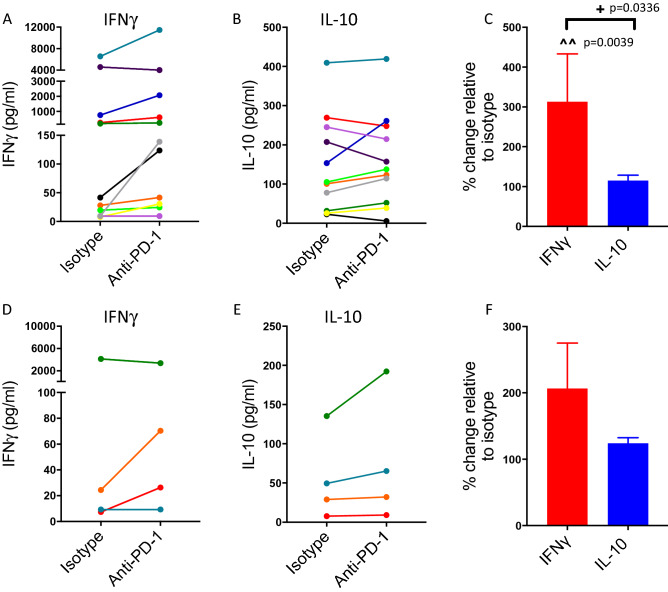
Response of dissociated tumour cells. Ability of pembrolizumab (anti-PD-1; 1.0 μg/ml) to modify IFNγ (**A**,**D**) or IL-10 (**B**,**E**) secretion by dissociated tumour cells from lung (**A**–**C**) or colon (**D**–**F**) tumours stimulated with allogeneic mature dendritic cells. (**A**,**B**,**D**,**E)** indicate responses of individual tumours (each colour represents an individual tumour), (**C**,**F**) indicate the percentage change in response in the presence of pembrolizumab relative to isotype control (mean + SEM; n = 11 [lung]; n = 4 [colon]). The impact of pembrolizumab was statistically significant compared to isotype control (^one sample Wilcoxon test). The difference between IFNγ and IL-10 was statistically significant (Mann–Whitney U test).

**Figure 6:**
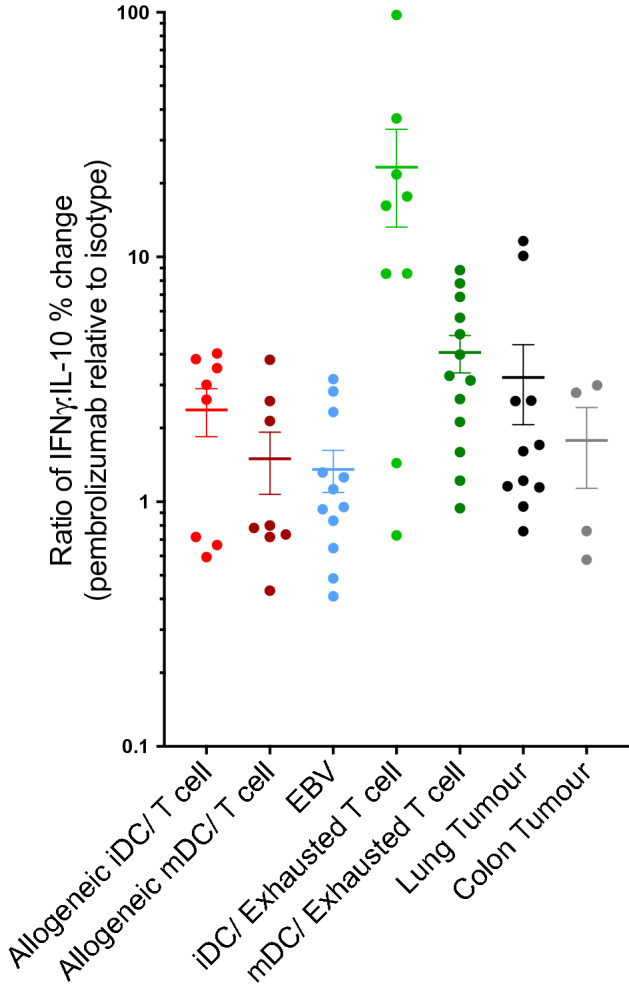
Ratio of IFNγ/IL10 responses across models. Graph showing the pembrolizumab-induced change in the ratio of IFNγ/IL-10 across the different in vitro models used in this study. Each point represents a donor/patient response. Mean ± SEM, n = 8 for allogeneic iDC or mDC/ T cell, n = 12 for EBV, n = 9–13 for iDC or mDC / Exhausted T cell, n = 11 for lung tumour and n = 4 for colon tumour.

To understand the impact of pembrolizumab in a clinical context, we cultured dissociated cells from solid tumours (either lung or colon tumours) with allogeneic DCs. Notably, pembrolizumab increased IFNγ levels in cultures from 9/11 lung carcinomas and 2/4 colon cancers (Fig. 5). The increase in IFNγ secretion from lung carcinomas elicited by pembrolizumab was statistically significant relative to isotype control (Fig. 5C). IL-10 levels by contrast were either not evidently affected or increased modestly only. In this context, as for the chronically activated T cells above, pembrolizumab skewed the cytokine response in favour of IFNγ over IL-10 (Fig. 6).

Interestingly, from the pathologist’s report, one of the lung tumours had a PD-L1 score of 55% (i.e. 55% of tumour cells demonstrated positive immunoreactivity for PD-L1 determined by immunohistochemistry) and the dissociated cells from this carcinoma demonstrated a particularly robust response (increased secretion of IFNγ and IL-10) to anti-PD-1 treatment in the presence of mature DCs (Supplementary Fig. S4 online) relative to isotype control. In contrast, such a response was not apparent in a separate lung carcinoma that displayed a PD-L1 score of < 1%. This supports a potential link between PD-L1 expression by lung carcinomas and the ability of dissociated cells to respond to PD-1 treatment although a greater number of cases would need to be evaluated to allow a confident conclusion.

## Discussion

Activation of T cells leads to the upregulation of inhibitory receptors including PD-1 and CTLA-4^10,11^. Chronic T cell activation, that may occur following repeated exposure to antigens in persistent infections or cancer, is also associated with increased expression of inhibitory receptors such as PD-1 and CTLA-4 as well as others but leads to a state of T cell dysfunction whereby productive T cell responses are compromised^12,13^. Canonical signalling via engagement with PD-1 renders the T cell receptor much less sensitive and inactivates CD28 signalling^14^. We have devised a number of in vitro models using human primary cells to study the functional impact of blocking PD-1 interactions with the clinically validated immuno-oncology therapeutic pembrolizumab (Keytruda). In all of the systems tested in this study, PD-1 blockade enhanced IFNγ production: albeit to varying degrees. This highlights the ability of PD-1 to impact both a polyclonal T cell population in the case of the allogeneic dendritic cell stimulation, and the response of chronically activated T cells to a recall antigen in the case of EBV peptide stimulation.

Consistent with the findings of others, pembrolizumab enhanced the increase in IFNγ levels-evoked by culture of allogeneic T cells with either immature or mature DCs across multiple donors^15^. Under these stimulation conditions, ipilimumab (Yervoy) did not enhance T cell proliferation or cytokine release. This may require titration of the dendritic cell/T cell ratio or may point to another mechanism of ipilimumab function within the tumour microenvironment, such as regulatory T cell depletion or other Fc-mediated mechanism^16–18^.

Following prolonged activation of T cells, there was an upregulation of PD-1, TIM-3 and CTLA-4, markers associated with T cell exhaustion, although a large elevation was not evident for all exhaustion markers studied (LAG-3 and TIGIT). Expression of LAG-3 demarked a population of cells that were predominantly positive for PD-1 and TIM-3. LAG-3 is an emerging checkpoint target for cancer immunotherapy and further testing the responses of this subpopulation of cells is a focus of our future studies^19^. Even without a complete complement of deemed exhaustion markers, culturing these chronically activated T cells with allogeneic dendritic cells greatly reduced IFNγ levels when compared to the response of non-stimulated (control) T cells. Strikingly, pembrolizumab was able to restore (mature DCs) or surpass (immature DCs) the IFNγ response of these chronically activated T cells. Again, despite the increase in CTLA-4 expression, under these conditions an impact of ipilimumab was not evident, which may be for the reasons discussed above. Although IL-10 levels were increased in the presence of pembrolizumab, they did not surpass those of control cells, suggesting that the response may be skewed towards a pro-inflammatory function. However, the role of IL-10 in anti-tumour immunity is complex with both tumour promoting and inhibiting actions demonstrated^9^. Still, the functional impact of IFNγ and IL-10 remain intriguingly linked as the anti-tumour functions of IL-10 appear dependent on IFNγ^20^. A consideration of this assay system is the potential presence of regulatory CD4^+^ T cells within the T cell population. These experiments were performed with the total CD4^+^ T cell population as we felt this better reflected the CD4^+^ T cell populations within the tumour and so potentially lead to a more predictive response. Mechanistic dissection of responses could be assisted by purification of subpopulations although an inhibition of PD-1 on regulatory T cells might be predicted to increase their suppressive function, which was not evident in this study, suggesting the majority of the response may be contributed by conventional CD4^+^ T cells^21^. Moreover, the activity of IFNγ in the tumour microenvironment is complex, with interferons produced by tumours conferring resistance to immune checkpoint blockade^22,23^. Indeed, disrupting the synthesis of interferons by tumours enhances the immune checkpoint blockade response^22,23^.

Pembrolizumab also enhanced intracellular expression of IFNγ and IL-10 by T cells following short-term stimulation with brefeldin ± PMA/ionomycin. It may be of interest in future studies to further phenotype the IFNγ positive cells with exhaustive cell markers (PD-1, TIM-3, LAG-3) to further investigate exhausted T cell sub-populations.

Although not as extensively studied as for CD8^+^ T cells, CD4^+^ T cells within the tumour environment do express markers of exhaustion^24^. In mouse lymphoma and solid tumour models, blockade of PD-1 can enhance CD4^+^ T cell-mediated anti-tumour immunity and in NSCLC patients, CD4^+^ immunity was required for clinical responses to PD-1/PDL-1 blockade therapy^25,26^.

Of all the in vitro models tested in the present study, the smallest pembrolizumab-mediated enhancement in IFNγ levels occurred in the PBMC culture stimulated with the EBV peptide pool. This system also showed minimal pembrolizumab skewing towards IFNγ over IL-10. The reduced potency of this system may reflect lower PD-L1 levels within the culture, or a reduced sensitivity to the PD-1/PD-L1 checkpoint for regulating EBV-specific T cell function. CD8-positive, EBV-specific, CD28-negative IFNγ-producing T cell populations have been described previously, and PD-1-mediated dephosphorylation of CD28 is therefore unlikely to affect these cells^14,27^. The presence of EBV-specific regulatory T cells, although not reported to produce IL-10, may further impact cytokine responses within this in vitro system^28^.

Many patients treated with checkpoint inhibitors do not respond clinically and there is also a risk of side effects. Only two out of four colon tumours tested in the present study responded to pembrolizumab treatment. Possible explanations for this lack of responsiveness include a lack or insufficient number of effector T cells in the tumour microenvironment, an exhausted T cell phenotype that could not be reversed by pembrolizumab, or additional suppressive factors secreted by tumour cells^29,30^. Of relevance, it is appreciated that biomarkers able to accurately predict patient responses would have significant clinical value^31^. Indeed, the FDA has approved IHC assays measuring PD-1 expression, and the expression of PD-L1 seems to be associated with enhanced responses to anti-PD-1/PD-L1 therapy^32^. Nevertheless, this approach still has significant limitations in predicting patients that will or will not respond to these therapies. Other approaches assessing soluble biomarkers, lymphocyte subsets and tumour immune infiltrate are also showing promise^24^. In vitro assays measuring production of soluble factors such as PGE2 , IL-6 or IL-10 by patient immune cells, T cell proliferation or STAT1 signalling may also have prognostic value in cancer^33–37^.

We found significant heterogeneity in anti-PD-1-induced increases (in terms of absolute levels or fold-changes in cytokines) and IFNγ/IL-10 ratios between cells from different patients or donors. In vitro assays using peripheral T cells or biopsy samples, such as those described in this study, could be explored for their value in predicting patient responses to PD-1/PD-L1 blockade, or, for identifying new therapeutic agents synergising with this pathway. In support of this, defects in pathways linked to the IFNγ-signalling appear involved in development of resistance to PD-1 blockade and subsequent studies have shown an IFNγ-related mRNA profile that may predict response to PD-1 blockade^38,39^. Evidence that peripheral CD8^+^ T cells proliferate following PD-1 therapy also supports the study of this accessible source of patient cells^40^. Key to evaluating the predictive value of these types of assay is matching the in vitro responses to clinical outcomes and therefore future studies would need to characterise carefully defined responder and non-responder patients. At present we acknowledge that the absence of this information is a limitation in our study. However, having now demonstrated potentially relevant in vitro assays, we will focus our attention to plan studies that although clearly technically challenging, and with a long path to clinical validation, assess whether in vitro assays such as those described in this study may be an essential component of providing affordable and personalised next-generation immuno-oncology therapies to the wider population.

## Supplementary Information

Supplementary Information.
