# Supplementary material for: Ex vivo modelling of PD-1/PD-L1 immune checkpoint blockade under acute, chronic, and exhaustion-like conditions of T-cell stimulation
Source: Sci Rep. 2021 Feb 17;11:4030. doi: 10.1038/s41598-021-83612-3 (PMC7889918; doi:10.1038/s41598-021-83612-3)
Supplement: Supplementary file 1 — Supplementary Information. [file 41598_2021_83612_MOESM1_ESM.pdf]

Ex vivo modelling of PD-1/PD-L1 immune checkpoint blockade under acute, chronic, and exhaustion-like conditions of T-cell stimulation

Alexander Roberts[1], Lindsay Bentley[2], Tina Tang[1,2], Fay Stewart[2], Chiara Pallini[2], Joel Juvvanapudi[2], Graham Wallace[3], Alison J. Cooper[1], Aaron Scott[3], David Thickett[3], Sebastian T. Lugg[3], Hollie Bancroft[7], Bridget Hemming[7], Charlotte Ferris[7], Gerald Langman[7], Andrew Robinson[7], Joanne Chapman[7], Babu Naidu[7], Thomas Pinkney[4], Graham S. Taylor[5], Kristian Brock[6], Zania Stamataki[5], Catherine A. Brady[2], S. John Curnow[2], John Gordon[2], Omar Qureshi[1,2], Nicholas M. Barnes[1*]

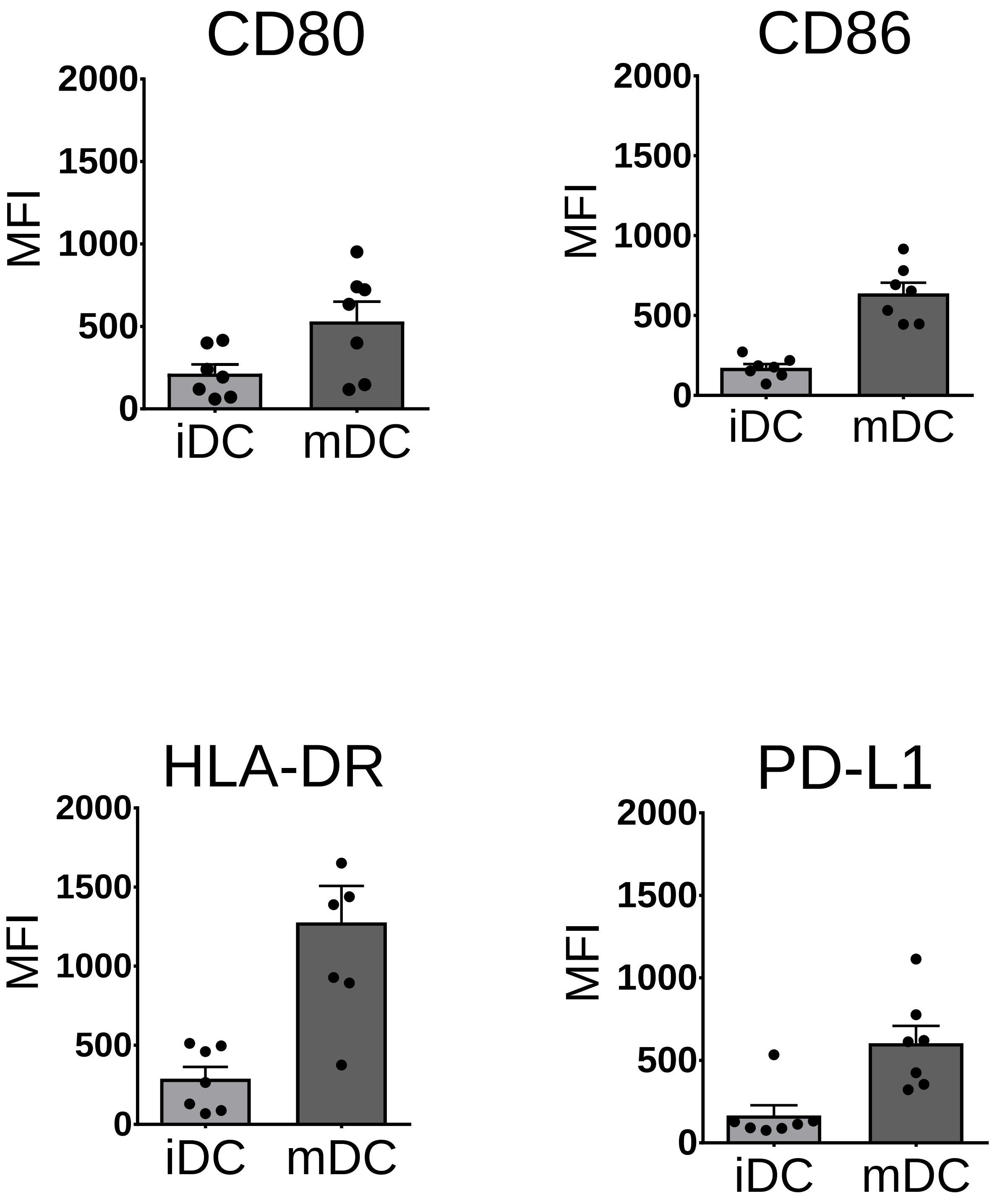

Supplementary Figure 1: Surface marker expression on mature dendritic cells
Maturation of dendritic cells (from immature [iDC] to mature [mDC]) is accompanied by an increase in expression of cell surface markers (CD80, CD86, HLA-DR and PD-L1). Data presented as mean fluorescent intensity (MFI), mean+SEM (and individual values), n=7.

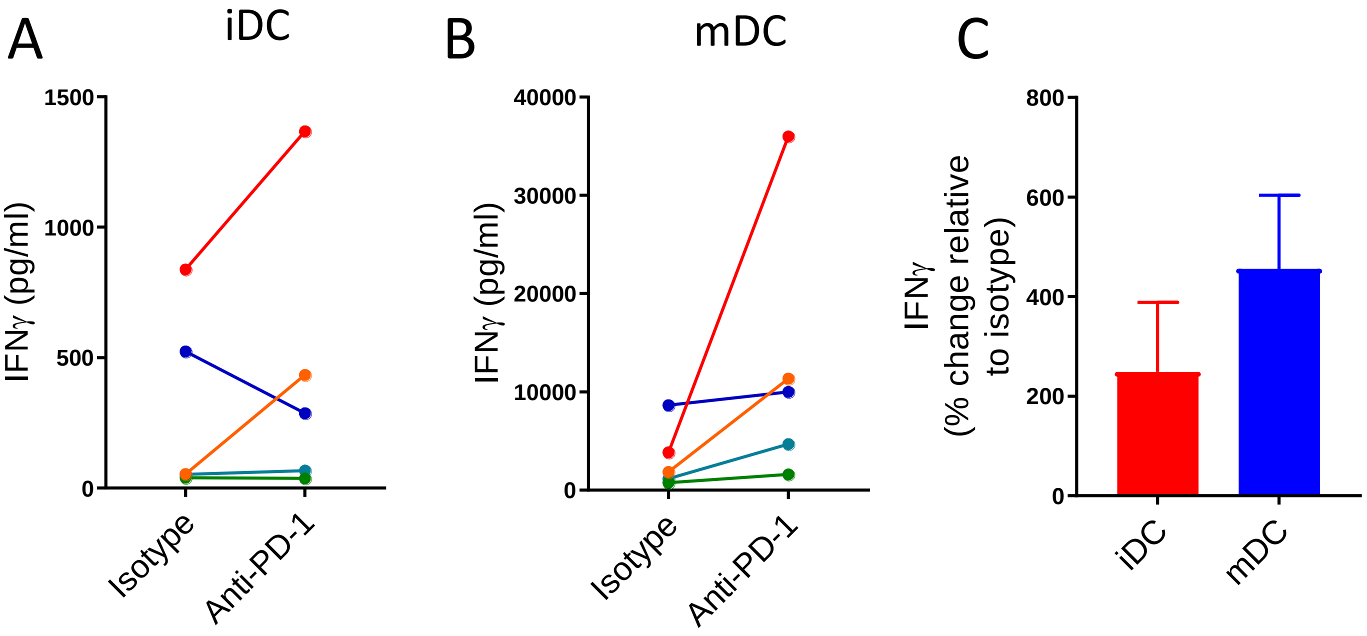

Supplementary Figure 2: Effects of pembrolizumab on purified CD8^{+} T cells in an allogeneic DC stimulation.

Ability of pembrolizumab (α-PD-1, 1.0 μg/ml) to modify IFNγ levels in co-cultures of CD8^{+} T cells and allogeneic immature or mature dendritic cells (iDC or mDC respectively) relative to isotype control. **A** and **B** demonstrate responses to individual donors (each colour represents an individual donor), **C** indicates percentage change in response in the presence of pembrolizumab relative to isotype control (mean+SEM, n=5).

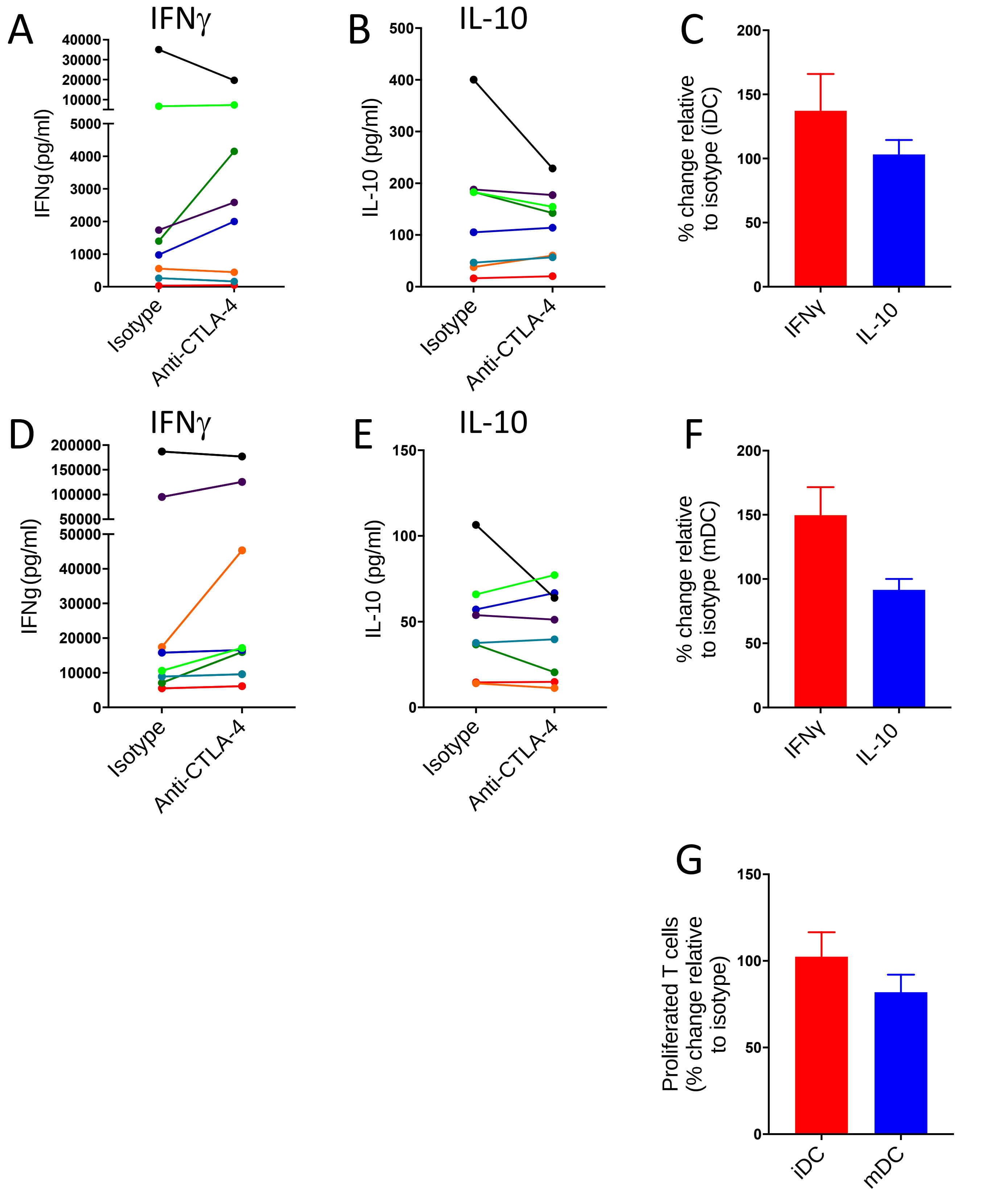

Supplementary Figure 3: Effects of ipilimumab on purified CD4^{+} T cells in an allogeneic DC stimulation.

Ability of ipilimumab (anti-CTLA-4, 1.0 µg/ml) to modify IFNγ (**A** and **D**) or IL-10 (**B** and **E**) levels in co-cultures of CD4^{+} T cells and allogeneic immature (**A - C**) or mature (**D - F**) dendritic cells relative to isotype control. **A**, **B**, **D** and **E** indicate responses of individual donors (each colour represents an individual donor), **C** and **F** show percentage change in response in the presence of ipilimumab relative to isotype control (mean+SEM, n=8). **G** Shows impact of ipilimumab upon CD4^{+} T cell proliferation in the presence of allogeneic immature or mature dendritic cells. Data presented as percentage change relative to isotype (mean+SEM, n=8).

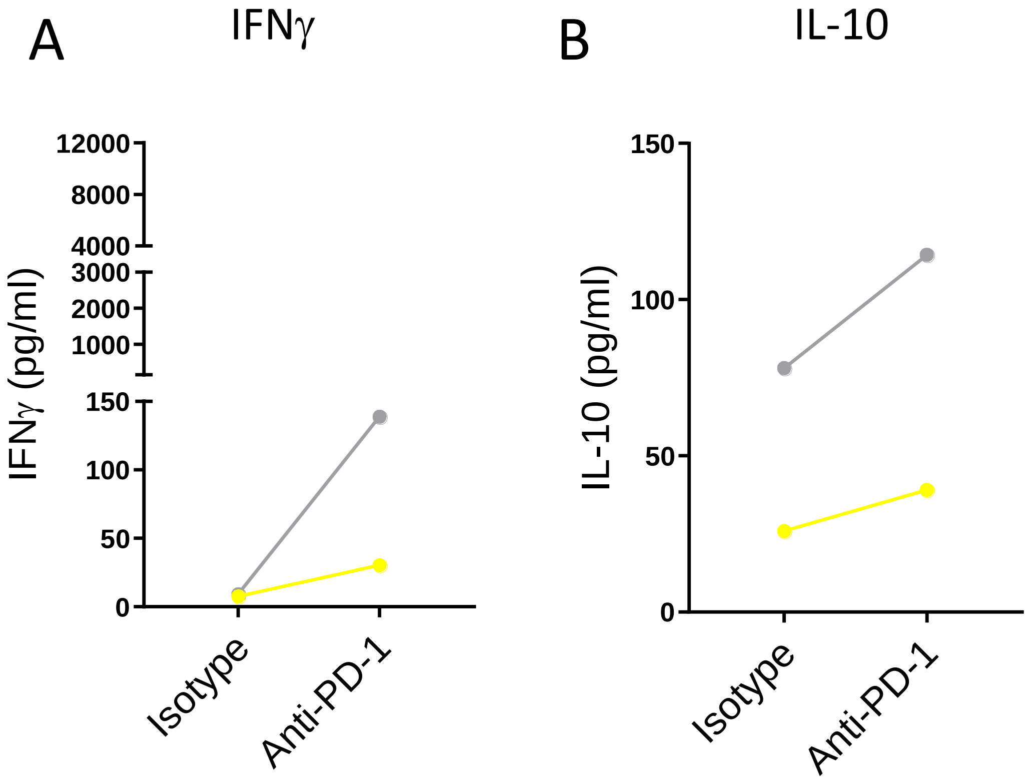

Supplementary Figure 4: PD-L1 scoring in lung tumours correlates with response to anti-PD-1 treatment.

Ability of pembrolizumab (anti-PD-1; 1.0 μg/ml) to modify IFNγ (**A**) or IL-10 (**B**) secretion by dissociated tumour cells from lung tumours stimulated with allogeneic mature dendritic cells. The patient represented by the grey line had a PD-L1 score of 55% (based on percentage of tumour cells expressing PD-L1 from pathologist's report) and showed a greater response (cytokine secretion) to pembrolizumab compared to the patient represented by the yellow line which had a PD-L1 score of < 1%, suggesting a correlation between PD-L1 expression by lung tumour cells and ability of infiltrating lymphocytes to respond to PD-1 treatment. n=2.

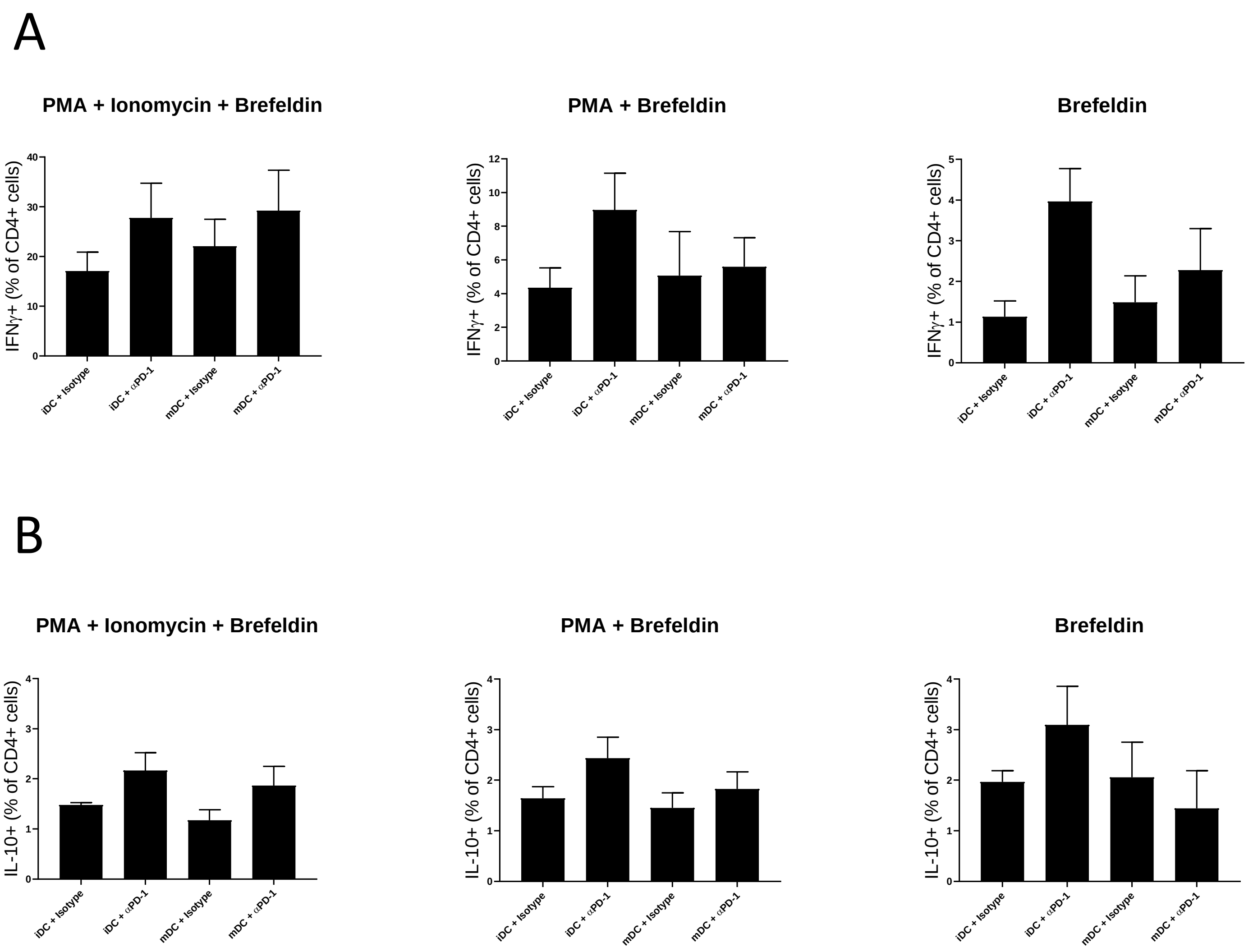

Supplementary Figure 5: Intracellular cytokine labelling of exhausted $CD4^+$ T cells.
Ability of pembrolizumab (α-PD-1; 1.0 µg/ml) to modify IFNγ (**A**) or IL-10 (**B**) expression following stimulation with PMA + Ionomycin + brefeldin, PMA + brefeldin or brefeldin alone in co-cultures of $CD4^+$ T cells pre-stimulated for 14 days with PHA (5.0 µg/ml) and allogeneic immature or mature dendritic cells (iDC or mDC) relative to isotype control. Data presented as percentage of IFNγ or IL-10 expressing $CD4^+$ T cells, mean+SEM, n=4.
